# Complete genome sequence analysis of *Edwardsiella tarda* SC002 from hatchlings of Siamese crocodile

**DOI:** 10.3389/fvets.2023.1140655

**Published:** 2023-03-09

**Authors:** Muhammad Nafees Ur Rehman, Farman Ullah Dawar, Jifeng Zeng, Lixia Fan, Wei Feng, Mengqi Wang, Nuo Yang, Guiying Guo, Jiping Zheng

**Affiliations:** ^1^Laboratory of Microbiological Engineering (Infection and Immunity), School of Life Sciences, Hainan University, Haikou, China; ^2^Department of Zoology, Kohat University of Science and Technology, Kohat, Pakistan; ^3^One Health Institute, Hainan University, Haikou, China

**Keywords:** *Edwardsiella tarda* SC002, genome annotation, pan-genome, genome sequencing, comparative genomics

## Abstract

*Edwardsiella tarda* is a Gram-negative, facultative anaerobic rod-shaped bacterium and the causative agent of the systemic disease “Edwardsiellosis”. It is commonly prevalent in aquatic organisms with subsequent economic loss and hence has attracted increasing attention from researchers. In this study, we investigated the complete genome sequence of a highly virulent isolate *Edwardsiella tarda* SC002 isolated from hatchlings of the Siamese crocodile. The genome of SC002 consisted of one circular chromosome of length 3,662,469 bp with a 57.29% G+C content and four novel plasmids. A total of 3,734 protein-coding genes, 12 genomic islands (GIs), 7 prophages, 48 interspersed repeat sequences, 248 tandem repeat sequences, a CRISPR component with a total length of 175 bp, and 171 ncRNAs (tRNA = 106, sRNA = 37, and rRNA = 28) were predicted. In addition, the coding genes of assembled genome were successfully annotated against eight general databases (NR = 3,618/3,734, COG = 2,947/3,734, KEGG = 3,485/3,734, SWISS-PROT = 2,787/3,734, GO = 2,648/3,734, Pfam = 2,648/3,734, CAZy = 130/3,734, and TCDB = 637/3,734) and four pathogenicity-related databases (ARDB = 11/3,734, CARD = 142/3,734, PHI = 538/3,734, and VFDB = 315/3,734). Pan-genome and comparative genome analyses of the complete sequenced genomes confirmed their evolutionary relationships. The present study confirmed that *E. tarda* SC002 is a potential pathogen bearing a bulk amount of antibiotic resistance, virulence, and pathogenic genes and its open pan-genome may enhance its host range in the future.

## Introduction

*Edwardsiella tarda* is a versatile bacterium that infects a broad range of organisms such as fish, amphibians, reptiles, birds, and mammals (1–3). *Edwardsiella tarda* infections show a broad spectrum of clinical manifestations in various organisms. For instance, Miyazaki and Kaige (4) reported necrotic lesions on multiple organs of Tilapia (*Tilapia nilotica*), Koeboelkuti et al. (5) reported multiple subcutaneous abscesses on grass snakes, while White et al. (6) reported hemorrhagic enteritis of pelicans. Some studies also observed septicemia and bacteremia in farmed hatchling crocodiles (7, 8), and meningitis, gastroenteritis, and soft tissue infections in humans (9, 10). Particularly, *E. tarda* is one of those pathogens infecting both cultured and wild aquatic species (3, 11). Hence, aquatic life is usually prone to acquiring *E. tarda* infections, and this fact has been extensively focused on to protect the aquaculture industry.

Studies on phenotyping, genotyping, and whole-genome sequencing reveal *E. tarda*'s diversity and pathogenicity (12, 13). The three genetically different taxa of *E. tarda* possess various degrees of pathogenicity in different hosts. Nearly all the fish pathogenic *E. tarda* isolates were acknowledged as *Edwardsiella anguillarum* and *Edwardsiella piscicida* (14, 15). Currently, the genus *Edwardsiella* of gamma *Proteobacteria* (16) consists of five species of *Edwardsiella*, namely, *tarda, hoshinae, ictaluri, anguillarum*, and *piscicida*. Particularly, *E. tarda* has the ability to attack epithelial cells (17, 18) and macrophages (19) where it multiplies intracellularly. It is one of the important steps of its pathogenesis by sabotaging the fish immunity and triggering systemic hemorrhagic septicemia (20). In addition, *E. tarda* uses numerous virulence factors such as secretion systems (T3SS and T6SS), adhesin, fimbrial adhesin-like protein (FimA), invasin (Inv), ferric uptake regulator (Fur), hemolysin (Hly), protease (PR), catalase (CAT), peroxidase (POD), and superoxide dismutase (SOD), siderophore, outer membrane proteins (OMPs), lipopolysaccharide (LPS), and two-component systems (TCSs) (3, 21).

From the economic point of view, the Siamese crocodile (*Crocodylus siamensis*) is a widely bred species in the South China region. A previous examination showed that immature Siamese crocodiles are vulnerable to genetic abnormalities, pilling up and asphyxia, mouth sores, skin diseases, gastropathy, and fungal and bacterial infections. Among bacterial infections, *E. tarda* was considered the main *Edwardsiella* species of septicemia among hatchlings and was controlled by the antibiotic “oxytetracycline” (7). However, later a new outbreak of Edwardsiellosis was reported and its subsequent investigation showed tetracycline-resistant *E. tarda* isolates (8). In the present study, from the aforementioned outbreak, a lethal isolate “*E. tarda* SC002” was subjected to whole-genome sequencing (8) and their genetic properties, virulence factors, invasive properties, and drug resistance were explored. The present study provides genomic insights into *E. tarda*, which will be indirectly helpful for candidate vaccine preparation against outbreaks of Edwardsiellosis in the aquaculture industry.

## Materials and methods

### Bacterial isolate and DNA extraction

*Edwardsiella tarda* SC002 was previously isolated from hatchlings of a diseased Siamese crocodile (8) and grown in BHI broth at 28 C overnight. After further sub-culturing up to the 40th generation, the bacterial genomic DNA from the exponential growth phase was extracted by the SDS technique. The extracted DNA was then subjected to 1% agarose gel electrophoresis for detection purposes and measured by a Qubit^®^ 2.0 Fluorometer (Thermo Scientific).

### Whole-genome sequencing procedures

The whole genome was sequenced using the Nanopore PromethION platform and the Illumina NovaSeq PE150 platform (Novogene Beijing, China). For sample preparation, 1 μg of DNA per sample was used as input material. According to the manufacturer's guidelines, sequencing libraries were prepared using NEBNext^®^ Ultra™ DNA Library Prep Kit for Illumina (NEB, USA). In brief, samples were sliced up to 350 bp by the sonication technique. The fragments were then end-polished, poly A-tailed, and ligated to a full-length adaptor for Illumina sequencing with further PCR amplification. Eventually, the amplified products were purified (AMPure XP system) and analyzed *via* Agilent2100 Bioanalyzer and quantified by real-time PCR. A hybrid assembly was generated using Unicycler software on short-read Illumina PE150 sequencing and long-read Nanopore sequencing data. The distribution of sequencing depth was counted, and the assembled sequence was specified as a chromosomal or a plasmid sequence according to size and alignment. The assembled sequence of whether it is a circular or a linear genome was also checked.

### Genome components' prediction

Various important components of the genome, including (CDSs) coding DNA sequences, repetitive sequences, non-coding RNAs (ncRNAs), genomic islands (GIs), transposons, prophages, and clustered regularly interspaced short palindromic repeat (CRISPR) elements, were predicted. The GeneMarkS package was used for retrieval of the associated coding gene.^1^ The RepeatMasker^2^ for the prediction of interspersed repeat sequences and the Tandem repeat finder for the detection of tandem repeats (22) were applied. tRNAscan-SE and rRNAmmer were used for the prediction of transfer RNA (tRNA) and ribosomal RNA (rRNA) genes, respectively (23, 24). The conserved small RNAs (snRNAs) were predicted by BLAST against the Rfam database (25). The IslandPath-DIOMB and the transposonPSI programs were applied to predict GIs, and transposons based on BLAST sequences, respectively (26). Furthermore, PHAST and CRISPRFinder were used for the identification of prophages and CRISPR elements, respectively.^3^

### Gene functional annotation

Some important databases for gene functional annotation, such as Gene Ontology (GO), Kyoto Encyclopedia of Genes and Genomes (KEGG), Clusters of Orthologous Groups (COG), Non-Redundant Protein Database (NR), Transporter Classification Database (TCDB), and SWISS-PROT databases, were used. A whole-genome BLAST with parameters such as *E*-value < 1e-5 and minimal identity > 40% was performed against the aforementioned seven databases. The secretory proteins and membrane proteins were predicted by the SignalP database and TMHMM Server, respectively.^4^ ^5^ In addition, type secretory proteins (types I–VII) were predicted by the EffectiveT3 tool.^6^ Gene clusters for secondary metabolism were evaluated by the antiSMASH tool (28). For the assessment of pathogenicity, the Pathogen–Host Interactions (PHI), the Virulence Factors Database (VFDB), and the Antibiotic Resistance Genes Database (ARDB) were used. Carbohydrate-active enzymes were identified by the Carbohydrate-Active enZYmes Database (CAZy).

### Phylogenomics and comparative genomics analysis

A genomewide comparison of the genus *Edwardsiella* members and species from related genera in the family Enterobacteriaceae, including the *Escherichia coli* (*E. coli*) strain 97-3250 (NZ_CP027599) and the *Salmonella enterica* strain FDAARGOS_878 (NZ_CP065718), was performed by Mauve genome alignment using the MegAlign_17 tool. A phylogenetic tree was constructed using the maximum-likelihood (ML) technique with the statistical support of 1,000 bootstrap replicates. Further analysis for carrying out comparative genomics was based on average nucleotide identity (ANI) values. The ANI values between the two genomes were calculated by the ANI calculator of Kostas lab (29) and visualized by a heatmap generated by the package ggplot2 in the statistical software R (version 2021-09.1+372). PGAweb, an online server, was used to estimate *E. tarda* pan-genome; however, incomplete genomes were excluded from the analysis (30).

## Results

### General characteristics of the whole-genome sequence

**Figure 1:**
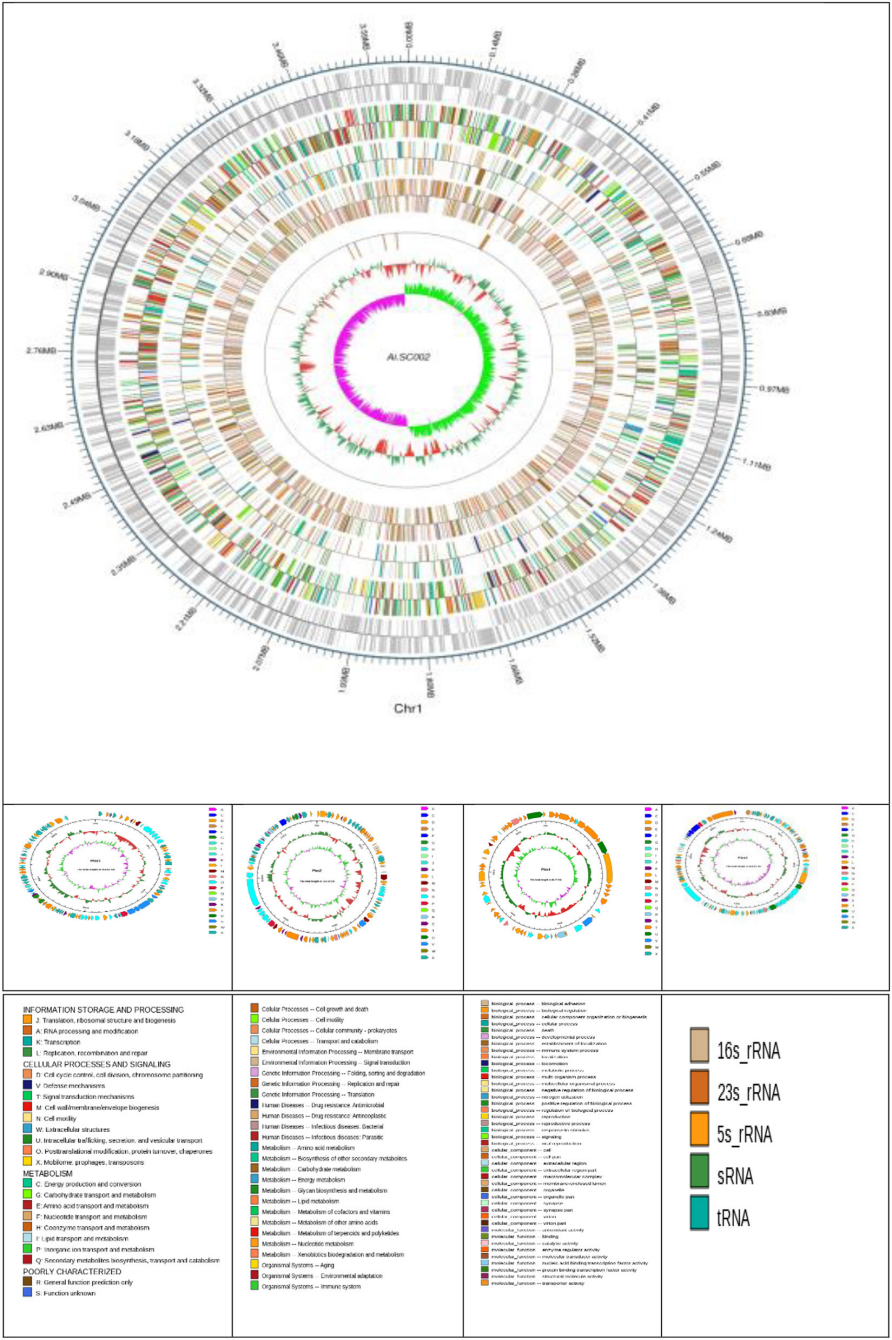
Atlas of the chromosome and four plasmids of *Edwardsiella tarda* SC002. From the outside to the center: genome sequence coordinates, genes encoded on forward and reverse strands, followed by COG annotation, KEGG annotation, GO annotation, ncRNA, GC content, and GC skew (G–C/G+C) GC/G+C.

*Edwardsiella tarda* SC002 is comprised of a circular chromosome of 3,662,469 bp with 57.29% of G+C content (Figure 1). The predicted genetic components are 3,734 genes, 12 genomic islands (GIs), a CRISPR component with a total length of 175 bp, and 106, 37, and 28 tRNAs, sRNAs, and rRNAs, respectively. Furthermore, 7 prophages (4 on chromosome and 3 on different plasmids), 48 interspersed repeat sequences (including LTR and one unknown repeat), and 248 tandem repeat sequences (including TRF, minisatellite, and microsatellite DNA) were predicted (Table 1). In addition, the predicted 3,734 coding DNA sequences (CDSs) have an average length of 916 bp, representing 82.83% of the total genome.

**Table 1:** Overall genomic features of the *Edwardsiella tarda* SC002.

**Attribute**	**Count/percent**
Chromosome	1 circular
Size	3,662,469 bp
G+C contents	57.29%
Genes	3,734
CRISPR	1
Genomic islands (GIs)	12
tRNA	106
rRNA	28
sRNA	37
Prophages	7
Interspersed repeat sequences	48
Tandem repeat sequences	248
Coding percentage	82.83%
**Plasmids**	4
pEtSC002-1	114,035 bp
G+C contents	52.11
Structure	Circular
pEtSC002-2	134,101 bp
G+C contents	48.82
Structure	Circular
pEtSC002-3	154,848 bp
G+C contents	50.15
Structure	Linear
pEtSC002-4	65,767 bp
G+C contents	51.46
Structure	Circular

### Conjugative plasmids

Four novel plasmids, namely pEtSC002-1, pEtSC002-2, pEtSC002-3, and pEtSC002-4, were observed, in which pEtSC002-1, pEtSC002-2, and pEtSC002-4 plasmids were circular with the sizes of 114,035 bp, 134,101 bp, and 65,767 bp, respectively, and pEtSC002-3 plasmid was linear with the size of 154,848 bp (Figure 1).

pEtSC002-1 encodes various Tra proteins (M, X, and Y domains*)*, replication initiation protein DnaC, and plasmid partition proteins (ParA *and* ParM), which show its resemblance to IncP plasmid. Additionally, the most abundant antibiotic resistance genes (*blaTEM, qnrS1, strB, sul2, aph3, sul1, cml*, and *tetA*) were found in the pEtSC002-1 plasmid.

pEtSC002-2 encodes a few Tra proteins (M, T, I, and Y domains), which indicate that it is a conjugative plasmid. However, plasmid partition proteins, such as SopA, SopB, and ParB, including plasmid replication initiator protein IncFII RepA, were encoded, indicating that it is an IncFII plasmid. There were no antibiotic resistance genes found in this plasmid; however, bacteriocin and colicin V were encoded which can act as efflux pumps conferring antibiotic resistance.

pEtSC002-3 was more unique than other plasmids observed in this study, a non-circular plasmid and one that encodes the maximum abundant number of Tra proteins (A, B, C, D, E, F, G, H, I, K, L, M, N, Q, T, U, V, W, X, and Y), indicating that it has a strong conjugation potential. Furthermore, plasmid partition proteins, such as ParA, ParB, and ParM, and a plasmid replication protein, DnaC of the IncFII RepA family, were encoded, indicating that it is also an IncFII plasmid.

pEtSC002-4 found in this study was a circular conjugative plasmid with various Tra (A, B, C, D, E, F, G, H, I, K, L, N, Q, T, U, and V) and Trb (B, C) proteins. The plasmid replication IncFII RepA protein family and ParA protein were found, indicating that it is an IncFII plasmid.

### Genomic plasticity and genomic islands

The G+C content shows high variability in the *E. tarda* SC002 genome (Figure 1). A significant part (213,631 bp; 5.17%) of the genome (4,131,220 bp) is comprised of genomic islands (GIs, *n* = 12), representing a complex structure of the genome. In addition, an adequate amount (*n* = 48) of interspersed repeat sequences (0.138% of 4,131,220 bp) and a variable number of tandem repeats (VNTRs) (*n* = 151, 0.435% of 4,131,220 bp) were detected. The GIs usually encoded hypothetical proteins, followed by the transposase, the integrase, the transcription regulator, transporter proteins, plasmid partition proteins, the terminase, phage proteins, and others (Table 2). The seven prophages (5.68% of 4,131,220 bp) were discerned, of which four were on a chromosome (average GC% = 53.19) and three were on two plasmids (average GC% = 50.56). Furthermore, prophages 4, 5, and 6 overlapped on several CDS regions of GIs 1, 4, and 5, respectively, on the chromosome. A valuable amount of ncRNA (1.3% of 4,131,220 bp) was observed as tRNA (*n* = 106), rRNA (*n* = 28), and sRNA (*n* = 37). A CRISPR component of 175 bp was found on plus strand of chromosome with 41 bp spacer and 31 bp direct repeat sequences (Table 1).

**Table 2:** Overview of the genomic islands in the *Edwardsiella tarda* SC002.

**GIs_id**	**Locus**	**CDSs**	**Function/characteristics**
GI001^a^	Chromosome	GM000423_GM000458	Hypothetical proteins, methyltransferase, lysozyme, terminase, T3SS
GI002	Chromosome	GM000461_GM000470	Phage integrase, hypothetical proteins, transcription regulator, T3SS
GI003	Chromosome	GM001349_GM001357	RNA helicase, hypothetical proteins, transposase for IS200, ribosome-binding ATPase, peptidyl-tRNA hydrolase, T3SS
GI004^b^	Chromosome	GM001632_GM001640	Phage protein, transposase for IS256
GI005^c^	Chromosome	GM001856_GM001912	Hypothetical proteins, antA/AntB antirepressor protein, phage protein, transcriptional regulator DNA helicase, replication protein, repressor, thymopoietin protein, excisionase, integrase, transposase, hydratase, DASS family transporter, sulfotransferase, lactoylglutathione lyase, antibiotic biosynthesis monooxygenase, T3SS
GI006	Chromosome	GM002263_GM002270	Hypothetical protein, NAD dependent epimerase, N-acetylmuramoyl-L-alanine amidase
GI007	Chromosome	GM002284_GM002299	Hypothetical protein, Nitroreductase, glutaredoxin 1, transport protein, integrase, transposes
GI008	Chromosome	GM002557_GM002592	Nucleoside transporter, peptidase C69 family, hypothetical protein, transcriptional activator, autotransporter, transposase InsB, single-strand binding protein, prophage protein, dipicolinate synthase, integrase, oxidoreductase, phosphate reductase, gamma-glutamyl kinase, Transcriptional regulator, T3SS
GI009	pEtSC002-2	GM003467_GM003474	Hypothetical proteins, resolvase, antitoxin RelB, plasmid-partitioning protein SopA
GI010	pEtSC002-2	GM003495_GM003509	Acyltransferase, filamentous hemagglutinin protein, transposase, hypothetical proteins,
GI011	pEtSC002-3	GM003626_GM003638	Outer membrane protein, pyrophosphorylase, transposase, hypothetical proteins, T3SS
GI012	pEtSC002-4	GM003691_GM003698	Hypothetical proteins, radical SAM superfamily, cloacin, transposase

a, b, c Genomic islands (GIs) are sharing coding sequences with the coding sequences of prophages 4, 5, and 6, respectively.

### Gene functional analysis

Gene functions were predicted by various databases such as COG, KEGG, GO, NR, Pfam, CAZy, and TCDB. The most abundant gene functions were predicted by NR (*n* = 3,618/3,734), followed by KEGG (*n* = 3,485/3,734), COG (*n* = 2,947/3,734), GO (2,648/3,734), and Pfam (2,648/3,734), TCDB (637/3,734), and CAZy (130/3,734).

**Figure 2:**
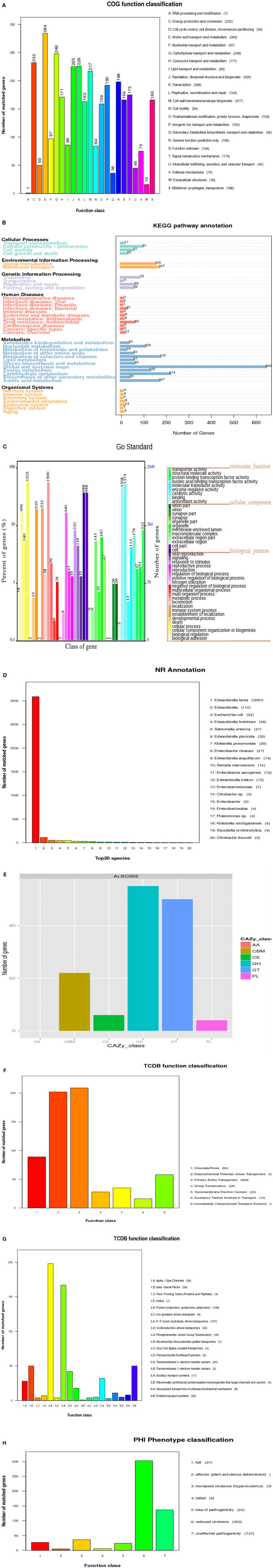
Gene functional annotation of the *Edwardsiella tarda* genome. **(A)** COG annotation distribution. **(B)** KEGG annotation distribution. **(C)** GO annotation distribution. **(D)** NR annotation distribution. **(E)** CAZy annotation distribution. **(F)** TCDB annotation distribution. **(G)** TCDB annotation distribution. **(H)** PHI annotation distribution.

Based on the orthology examination, the COG database predicted 2,947 genes (78.92%), and these genes were distributed into 23 functional categories (Figure 2A). According to the COG categorization, the five most rich annotated functions were amino acid transport and metabolism (284 genes), carbohydrate transport and metabolism (248 genes), energy production and conversion (232 genes), transcription (226 genes), and translation, and ribosomal structure and biogenesis (225 genes). Furthermore, 166 hypothetical genes were discerned, which may need to be explored by further studies. In addition, 16 genes for extracellular structures, 166 genes each for transposons and prophages, and one gene were found for RNA processing and modification (Supplementary Tables S1, S2; Figure 2A).

The KEGG database annotation represents a total of 2,510 genes. Followed by the classification of the KEGG orthology (KO) database, the annotated genes were distributed into six categories: metabolism (1,663), environmental information processing (317), genetic information processing (190), cellular processes (175), human diseases (126), and organismal systems (39) (Figure 2B). According to the orthology results, the most populated class was represented by metabolic pathways, global and overview maps from the “Metabolism” category, with 587 genes. The second abundant class was the biosynthesis of secondary metabolites from the “Metabolism” category (267 genes), followed by microbial metabolism in diverse environments (184 genes) and biosynthesis of antibiotics (175 genes) from the “Metabolism” category. In addition, two-component systems (TCSs) and ABC transporters were discerned with 138 and 123 genes, respectively, from the “Environmental Information Processing” category (Supplementary Tables S1, S2).

According to GO analysis, a total of 2,648 protein-encoding genes were annotated (Figure 2C). The annotated genes were mainly categorized into 43 subfunctional items. These were further distributed into three major categories: biological process (22 subfunctions), cellular component (12 subfunctions), and molecular function (9 subfunctions). However, a total of 1,525 different genes, followed by 1,500, and 532 belonging to the cellular process, metabolic process, and localization, respectively, of the biological process category were annotated (Supplementary Tables S1, S2).

According to the NR database, a total of 3,618 genes were annotated within 80 different bacterial species. The top 20 species in which *E. tarda* have 3,087 genes were followed by *Edwardsiella* species, in which *Edwardsiella* has 110 genes, *Escherichia coli* (NZ_CP027599) has 52 genes, *Edwardsiella hoshinae* (*E. hoshinae*) (NZ_CP065626) has 48 genes, and *Salmonella enterica* (CP006631) has 47 genes, respectively (Supplementary Tables S1, S2; Figure 2D).

The Pfam database analysis annotated a total of 2,648 genes, which are grouped into 294 clans. The five most abundant gene containing clans were cl0023.33 followed by cl0063.24, clan0123.17, cl0344.3, and cl0015.19 with 1,041, 675, 514, 361, and 92 genes, respectively (Supplementary Tables S1, S2).

The CAZy analysis shows that 130 genes are matching with 59 CAZy-family genes. The CAZy families GT2, GH23, and CMB50 have the most abundant number of genes, which were 14, 13, and 12, respectively. However, the CAZy class annotation shows that GH, GT, and CBM have 55, 50, and 22 matching genes (Supplementary Tables S1, S2; Figure 2E).

Transporter Classification Database (TCDB) annotated a total of 637 genes and categorized them into 7 classes and 18 subclasses. Class 3 has the highest number of genes followed by class 2 and class 1 with 209, 202, and 89 genes, respectively (Figure 2F). The subclassification revealed that the highest number of genes belong to subclass 2.A, followed by 3.A, 1.B, 9.B, and 3.D with 198, 167, 50, 50, and 42 genes, respectively (Supplementary Tables S1, S2; Figure 2G).

### Pathogenicity/virulence factor analysis

To evaluate the pathogenicity of SC002, databases, such as ARDB, CARD, PHI, and VFDB, were searched. According to Antibiotic Resistance Genes Database (ARDB) analysis, 11 genes were annotated, of which two each of tetracycline, streptomycin, and sulfonamide resistance and 1 each of aminoglycosides, bacitracin, penicillin, fluoroquinolone, and chloramphenicol resistance were found (Supplementary Table S3). The Comprehensive Antibiotic Resistance Database (CARD) analysis annotated a total of 142 genes, in which *macB* was the most prevalent, with eight genes followed by five *evgS* and four each of *gadX* and *TaeA* genes (Supplementary Tables S1, S3). The pathogen–host interactions database (PHI) annotates a total of 538 genes categorized into six classes, of which the most populated one was “reduced virulence”, followed by “unaffected pathogenicity” and “hypervirulence” with 303, 137, and 36 genes, respectively (Figure 2H). The Virulence Factor Database (VFDB) detected a total of 315 genes, and the most common genes were related to virulence factors such as “flagellar protein,” “LOS,” and “capsule” (Supplementary Tables S1, S3).

### Effector/secretory protein profiling

**Figure 3:**
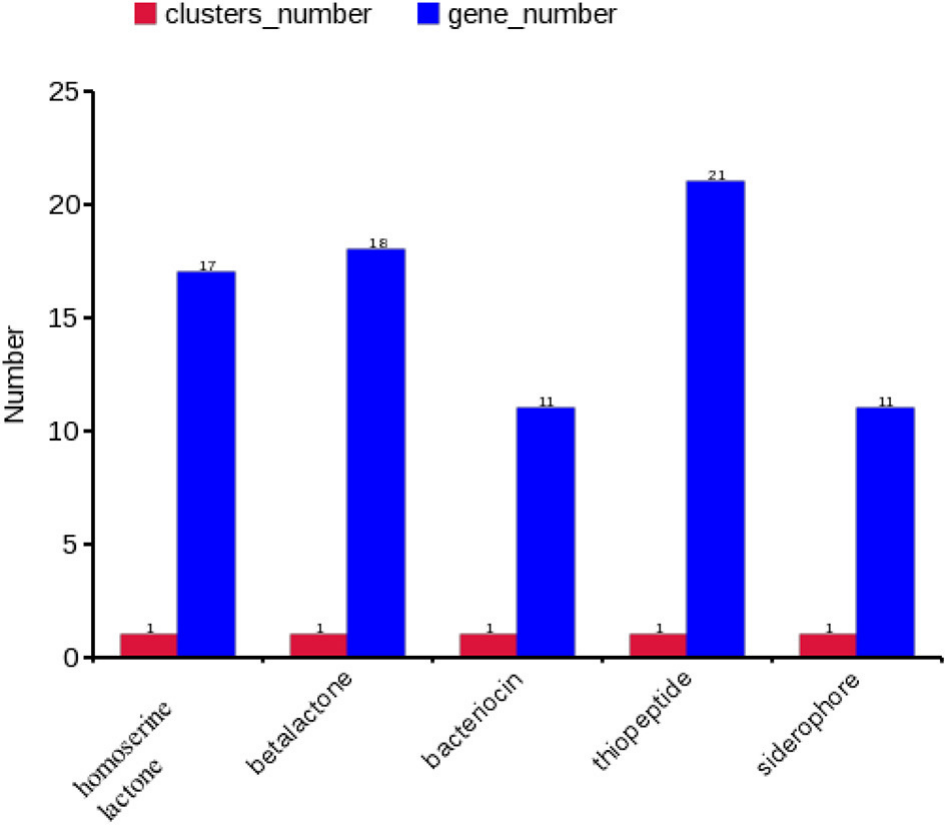
Secretory protein annotation of an SC002 genome. Five clusters with their respective numbers of genes.

A gene cluster is a pool of genes on DNA encoding similar proteins. These proteins collectively perform a generalized function. Gene cluster size is inconsistent, from a few genes to some 100 genes (31). For secondary metabolite evaluation, there are five clusters encoded by the genome. Cluster 1 was identified as homoserine lactone that consists of 17 genes, Cluster 2 was betalactone with 18 genes, Cluster 3 was bacteriocin with 11 genes, Cluster 4 was thiopeptide with 21 genes, and Cluster 5 was siderophore with 11 genes (Figure 3). Furthermore, Clusters 1–4 were encoded by chromosome, while Cluster 5 was present on pEtSC002-4 (Table 3). All of the aforementioned clusters have 100% homology with reference species, except Cluster 4, which has 33% homology. Upon secretory protein analysis, 209 gene-encoding proteins were predicted as signaling proteins. Furthermore, 129 genes were predicted for encoding T3SS proteins, of which 16 genes were encoded by all the plasmids.

**Table 3:** Effector/secretory protein profiling encoded by the genome.

**Clusters**	**Genes**	**Locus**	**Homology (%)**
Homoserine lactone	17	Chr	*E. tarda* FL95-01 (100%)
Betalactone	18	Chr	*E. tarda* ATCC 15947 (100%)
Bacteriocin	11	Chr	*E. tarda* ASE201307 (100%)
Thiopeptide	21	Chr	*Serratia fonticola* AU-P3(3) (33%)
Siderophore	11	pEtSC002-4	*E. tarda* ATCC 15947 (100%)

### Phylogenetics and comparative genomics analysis

**Figure 4:**
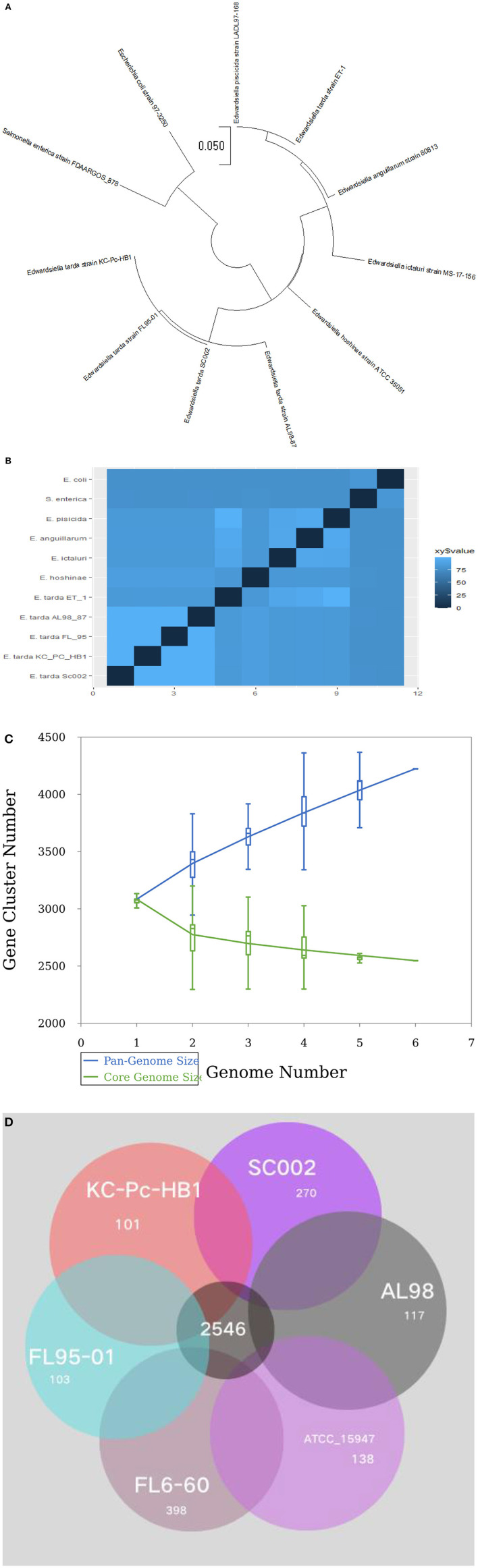
Phylogenetic and comparative genomics analysis: **(A)** A total of 11 genomes of *Edwardsiella* species, including two outgroup organisms—*Salmonella enterica* and *Escherichia coli*, were analyzed by the Mauve algorithm and a phylogenetic tree was constructed based on conserved aligned blocks of complete genomes. **(B)** A heatmap of 11 genomes based on the average nucleotide identity (ANI) values between two species. **(C)** A line chart represents an open pan-genome of six *Edwardsiella tarda* strains. **(D)** Venn diagram of core and unique genes shared by a comparison of six known *E. tarda* strains.

For evolutionary relationships, SC002 and 10 strains of the genus *Edwardsiella* including two outgroup organisms were compared. A phylogenetic tree was constructed based on conserved aligned blocks, which shows a clearly divergent lineage. Four *E. tarda* strains were clustered together and one strain of *E. tarda* was branched with *E. piscicida* species (previously known as *E. tarda*) (Figure 4A). To further explore the evolutionary relationships, ANI values were discerned. The ANI values were compared to estimate genomic differences and relatedness between two genomes. According to our results (Figure 4B), the genomes of four *E. tarda* strains shared ANI values ranging from 99.34 to 99.45%. These values are above the threshold of 94–96% identity, which is usually considered a speciation edge (32). However, the strain ET_1 shared ANI values of 83% with *E. tarda* strains and 99.85% with *E. piscicida* strains (formerly known as *E. tarda*), which clearly shows that it is a distinct species currently classified as *E. tarda*.

Pan-genome analysis for six *E. tarda* strains (Figure 4C), shows that the species have an open pan-genome. It is comprised of 4,222 cluster genes, distributed into 2,546 core cluster genes (60%), 558 accessory genes (13.2%), and 1,118 unique genes (26.5%). A number of variable genes ranging from 101–398 were recognized in the unique genes. The maximum and minimum numbers of unique genes were observed in the *E. tarda* strains of FL6-60 (NC_017309) and KC_Pc_HB1 (NZ_CP023706), respectively (Figure 4D). Notably, our strain SC002 contained 270 unique genes (Figure 4D) and shares a higher number of core (conserved) genes with strains ATCC_15947 (2639), FL95-01 (2637), FL6-60 (2634), AL98 (2633), and KC_PC_HB1 (2631). Collectively, these results indicated that the genome structure of the six *E. tarda* strains has high conservation and diversity.

## Discussion

Edwardsiellosis is a global issue for the aquaculture industry in the current era. Therefore, an understanding regarding the genetic makeup of *Edwardsiella* is needed that may be helpful in finding ways to overcome their outbreak in the aquatic species. Accordingly, this study isolated *E. tarda* SC002, a virulent strain from Siamese crocodile hatchlings in the Hainan Province of China, and their whole genome was sequenced to determine its pathogenicity, virulence, and niche-related properties. The SC002 genome has relatively higher coding and non-coding genes (tRNA, rRNA, and sRNA) than the other sequenced *Edwardsiella* species (such as KC_Pc_HB1, C07-087, and FL95-01) and is relevant to the rapid growth of the bacterium (33). The 5S rRNA gene was found to double in our study, which is similar to that mentioned in a previous study (34). Previously, the isolate SC002 was clustered with ATCC 15945 (8), indicating that it is a genotype of mammalian origin (13, 35). The G+C content of SC002 was 57.29%, which is similar to a previous study where the G+C content of *Edwardsiella* genomes ranges from 56.8 to 59.80% (36).

Unlike the published *Edwardsiella* complete genomes having one or two plasmids, the SC002 genome contained four novel plasmids (34, 37–39). Subsequently, BLAST analysis shows that the pEtSC002-1 was identical to the *S. enterica* plasmid (NZ_CP037959.1), while pEtSC002-2, pEtSC002-3, and pEtSC002-4 were identical to the *E. tarda* KC_PC_HB1 plasmid (NZ_CP023707.1) (40, 41). The antibiotic resistance determinants were only observed on pEtSC002-1 where the resistance gene patterns (*sul1, sul2, cml, tetA, aph33*, and *aph6*) were similar to those of EIB202 plasmid resistome (33). The β-lactam and fluoroquinolone resistance genes were additional to pEtSC002-1, indicating that this plasmid is more virulent than the latter plasmid. Three T3SS genes were annotated on pEtSC002-1, whereas plasmid pEIB202 encodes an incomplete set of T4SS genes which may play a role in horizontal gene transfer (HGT) (34). However, the detection of two prophages on pEtSC002-1 may enhance its capability for producing genetic exchange among bacteria. Furthermore, all plasmids share conjugative plasmid features and encode for Tra, replication initiation, and plasmid partition proteins, which shows that these plasmids might belong to the IncP plasmid that was capable of undergoing replication and stable inheritance in a wide variety of Gram-negative bacteria (42).

According to our study, 12 genomic islands (GIs) were detected in SC002, whereas 11 in *E. tarda* FL6-60, 24 in *E. piscicida*, and 31 in *E. ictaluri*, which shows that the taxawise GIs are frequent as *E. tarda* < *E. piscicida* < *E. ictaluri* (34). However, 5 out of 12 GIs of SC002 encode for T3SS proteins, whereas in EIB202, two GIs: GI7 and GI17 encode for T3SS and T6SS, respectively (34), which shows that these GIs contribute to the pathogenicity of SC002.

The analysis of the SC002 genome suggests that it has the ability to deal with environmental changes due to the presence of gene regulation systems, such as two-component systems (TCSs) and quorum sensing systems. In the genome of SC002, a total of 138 TCS genes and 50 quorum-sensing genes were detected, which were more than those of the previously reported *E. piscicida* (34). Usually, TCS genes are adjacent; however, similar to a previous study (34), the barA/UvrY, arcA/B, and cheA/B/Y, TCSs in the present study are unusually organized. The presence of some well-known TCSs explored previously in *E. tarda*, such as PhoP/Q (43) and QseB/C (44), might have a role in the pathogenicity and virulence of SC002.

*Edwardsiella tarda* is capable of surviving and persisting intracellularly in phagocytes followed by systemic infection (45). To resist phagocyte-mediated killing, various essential strategies, such as enzyme production including CAT, POD, and SOD, have been implicated by *E. tarda* to neutralize reactive oxygen species (ROS), while TTSS and T6SS contribute to invasion and subversion of the host cells (46). In the present study, 129 T3SS genes were predicted; however, in a previously reported *E. tarda* KC-Pc-HB1, no T3SS/T6SS genes were detected (41), indicating that SC002 is a more virulent strain.

A previous study revealed the mechanism of *E. tarda* host adaptation and interspecies discrepancies within the species of the genus *Edwardsiella* (35). Phylogenomics and pairwise ANI comparison show that the *E. tarda* strain ET_1 was clustered to *E. piscicida*, calling for reconsideration of the genus *Edwardsiella*. Horizontal gene transfer (HGT) in *E. tarda* was responsible for the acquisition of the locus of enterocyte effacement (LEE) (47). Host specificity in *Staphylococcus aureus* was associated with host-adaptive gene transformation *via* mobile genetic elements (MGEs) (48). According to the present study, *E. tarda* contains an open pan-genome, which is congruent with a previous study (49). Consequently, the pathogen has a chance to expend its host niches in the future. Further study is needed to experimentally prove the function of unique genes in host specificity and evolution. *E. tarda* is a well-known aquatic pathogen affecting the aquaculture industry worldwide. Its target is not only to attack high-value fish species such as turbot but also to inflict injuries on birds, reptiles, and mammals. Therefore, strict measures to control the pathogen and proper vaccine candidate development are needed.

## Conclusion

The whole-genome sequence of SC002 was characterized as highly virulent and multidrug resistant (MDR). A thorough examination of the genome sequence shows that the pathogen has an array of drug-resistant genes on pEtSC002-1. The conjugative and prophage determinants of the observed plasmids further considered that the contents of the genome are partly structured in various aquatic ecological niches during its life cycle. An adequate amount of TCSs' quorum sensing and T3SS genes that showed the diverse nature of the pathogen were confirmed. By exploring the understanding of the pathogenesis, virulence, and host specificity of the organism and by adopting the approach of “reverse vaccinology”, this study lays the foundation for candidate vaccine development.

## Data availability statement

The complete genome sequence of the Edwardsiella tarda SC002 chromosome and the plasmids pEtSC002-1, pEtSC002-2, pEtSC002-3, and pEtSC002-4 have been deposited to the GenBank under accession numbers CP116675, CP116676, CP116677, CP116678, and CP116679 respectively.

## Ethics statement

The animal study was reviewed and approved by Hainan University Laboratory Animal Care Committee (HULACA180703). Written informed consent was obtained from the owners for the participation of their animals in this study.

## Author contributions

All authors listed have made a substantial, direct, and intellectual contribution to the work and approved it for publication.

## Footnotes

^1^ http://topaz.gatech.edu/

^2^ http://www.repeatmasker.org/

^3^http://phast.wishartlab.com (27).

^4^ https://services.healthtech.dtu.dk/service.php?SignalP-5.0

^5^ https://services.healthtech.dtu.dk/service.php?TMHMM-2.0

^6^ https://www.effectivedb.org/method/effectivet3
